# *Pseudanthiashangapiko*, a new anthiadine serranid (Teleostei, Serranidae, Anthiadinae) from Rapa Nui (Easter Island)

**DOI:** 10.3897/zookeys.1054.64508

**Published:** 2021-08-03

**Authors:** Bart Shepherd, Hudson T. Pinheiro, Tyler A. Y. Phelps, Alejandro Pérez-Matus, Luiz A. Rocha

**Affiliations:** 1 Steinhart Aquarium, California Academy of Sciences, San Francisco, CA 94118, USA Steinhart Aquarium, California Academy of Sciences San Francisco United States of America; 2 Department of Ichthyology, California Academy of Sciences, San Francisco, CA 94118, USA Department of Ichthyology, California Academy of Sciences San Francisco United States of America; 3 Department of Biology, San Francisco State University, San Francisco, CA 94132, USA San Francisco State University San Francisco United States of America; 4 Subtidal Ecology Laboratory, Estación Costera de Investigaciones Marinas, Facultad de Ciencias Biológicas, Pontificia Universidad Católica de Chile, Casilla 114-D, Santiago, Chile Pontificia Universidad Católica de Chile Santiago Chile; 5 Millennium Nucleus for the Ecology and Conservation of Temperate Mesophotic Reef Ecosystem (NUTME), Las Cruces, Valparaíso, Chile Millennium Nucleus for the Ecology and Conservation of Temperate Mesophotic Reef Ecosystem Las Cruces Chile

**Keywords:** Biodiversity, coral-reef twilight zone, ichthyology, island, mesophotic coral ecosystem, reef fish, taxonomy

## Abstract

*Pseudanthiashangapiko***sp. nov.** (Teleostei, Serranidae, Anthiadinae) is herein described from three specimens collected from a depth of 83 m in a mesophotic coral ecosystem off Hanga Piko, Rapa Nui (Easter Island), Chile. *Pseudanthiashangapiko***sp. nov.** can be distinguished from its congeners in live coloration and by the following combination of characters: dorsal-fin rays X, 17; anal-fin rays III, 8; pectoral-fin rays 16 (left side of one specimen 17); vertebrae 10+16; scales relatively large, two scales above lateral-line to base of fifth dorsal spine, and 16–17 circumpeduncular scales; gill rakers 11+23; and a slender body, with greatest body depth 3.6 (3.4–3.8) in SL. The most similar DNA barcodes (mitochondrial COI gene) are from *Pseudanthiasventralis* Randall, 1979 and *Pseudanthiashawaiiensis* Randall, 1979, with 16.8% and 17.0% uncorrected divergence, respectively. This fish is one of four new species that were documented from a pair of technical dives at a single location in Rapa Nui, emphasizing the high number of undescribed species likely still unknown in mesophotic coral ecosystems, especially in geographically remote locations. *Pseudanthiashangapiko***sp. nov.** adds to the Rapa Nui ichthyofauna, which hosts the second-highest level of endemism in both shallow and deep-water fishes.

## Introduction

The genus *Pseudanthias* Bleeker, 1871 (Teleostei, Serranidae, Anthiadinae) currently comprises more than sixty valid species found globally in temperate and tropical oceans (Allen and Erdmann 2012). Many members of the genus are protogynous hermaphrodites, and most can be easily identified by the sexually dichromatic coloration of living adult specimens (Kuiter 2004). They are conspicuous inhabitants of coral reef environments and mesophotic coral ecosystems (**MCEs**), occurring in large haremic aggregations and feeding on zooplankton (Allen and Erdmann 2012). The highest diversity of *Pseudanthias* species is recorded in the coral triangle, with nearly 30 described species occurring in Indonesia and the Philippines alone (Allen and Erdmann 2012). Moving eastward out of the coral triangle and into the South Pacific, diversity within the genus decreases, and the relative proportion of endemic species increases, following the general pattern observed in other groups (Randall 1998; Allen and Erdmann 2012). Most *Pseudanthias* species occur on MCEs (Rocha et al. 2018), where several new species have been recently described (Gill et al. 2017; Gill and Psomadakis 2018; Victor et al. 2020), a trend in discoveries that results from increased exploration of the deeper sections of coral reef habitats (Rocha et al. 2018; Pinheiro et al. 2019; Pimentel et al. 2020).

Through a partnership between the Pontificia Universidad Católica de Chile and the Hope for Reefs Initiative of the California Academy of Sciences, our team conducted surveys using technical diving and closed-circuit rebreathers (Hollis Prism 2) to depths of 110 m to identify and document the fish communities associated with MCEs at Rapa Nui. Here we describe a new species of *Pseudanthias* collected in a MCE off Hanga Piko, Rapa Nui (Easter Island), Chile in March 2017. This is the fourth species description resulting from this expedition to Rapa Nui, and the sixth member of the subfamily Anthiadinae to be found at the island. *Caprodonlongimanus* Günther, 1859, *Plectranthiasparini* Anderson & Randall, 1991, *P.ahiahiata* Shepherd, Phelps, Pinheiro, Pérez-Matus & Rocha, 2018, and *Luzonichthyskiomeamea* Shepherd, Pinheiro, Phelps, Pérez-Matus & Rocha, 2019 are the other described Anthiadinae known from Rapa Nui; a presumed *Tosanoides* has also been observed with remotely operated vehicles but has yet to be captured and examined (Easton et al. 2017). These species, along with *Chromismamatapara* Shepherd, Pinheiro, Phelps, Easton, Pérez-Matus & Rocha, 2020, and the new anthias presented here, appear to be restricted to MCEs, as is the case for much of the ichthyological biodiversity being revealed by scientists exploring these ecosystems (Rocha et al. 2018; Pinheiro et al. 2019).

## Materials and methods

Three individuals of the new species were collected with hand nets while diving on mixed-gas, closed-circuit rebreathers (Hollis Prism 2) around Rapa Nui, in March 2017. Specimens were collected and immediately transported to a field laboratory where they were photographed, tissue sampled, fixed in 10% formalin, and preserved in 75% ethanol. Measurements and x-radiographs were made at the California Academy of Sciences three and one-half years later. Counts were performed with the aid of a stereomicroscope. Morphological characters were measured to the nearest 0.01 mm with digital calipers following the conventions described in Anderson et al. (1990), Anderson and Heemstra (1980, 2012), and Gill et al. (2017). Morphometric data for the holotype and paratypes are presented in Table 1. Proportional measurements in the text are rounded to the nearest 0.1 mm. We measured standard length (SL) as the straight-line distance from the anteriormost point of the upper lip to the base of the caudal fin. Head length (HL) was measured from the anteriormost point of the upper lip to the posterior end of the opercular flap. Body depth (BD), was measured as the maximum depth from the origin of the spinous dorsal fin. Counts of principal caudal-fin rays follow Gill et al. (2016), and are presented in the form upper + lower, where the upper rays are those associated with hypurals 3–5, and the lower rays are those associated with hypurals 1–2 and the parhypural. Procurrent caudal-fin rays are those dorsal and ventral to the principal rays. Elongated filaments on the fin rays were not included in the measurements. Vertebral counts include the last vertebra fused to the hypural plate; vertebral counts are presented as precaudal + caudal, where the first caudal vertebra is the anteriormost vertebra bearing a haemal spine. Gill raker counts were made on the left side first arch, and are presented as upper (epibranchial) + lower (ceratobranchial) rakers on the anterior face of the first arch; the angle raker is included in the lower limb count. In the description, data are given first for the holotype, followed by a range of values for the paratypes, in parentheses, where variation was noted. Where counts are recorded bilaterally, both counts are given and separated by a vertical line (|). Where there is some degree of uncertainty, counts are followed by a question-mark (?). Comparisons with related species were based on literature accounts, particularly morphometric and meristic data from *Pseudanthiasventralis* Randall, 1979 and *Pseudanthiashawaiiensis* Randall, 1979, the more recently described *Pseudanthiastequila* Gill, Tea & Senou, 2017, *Pseudanthiasemma* Gill & Psomadakis, 2018, *Pseudanthiastimanoa* Victor, Teitelbaum & Randall, 2020, Allen and Erdman (2012), and Anderson’s (2018) checklist of the Anthiadinae.

**Table 1.** Morphometric data for the three specimens of *Pseudanthiashangapiko* sp. nov. expressed as percentage of standard length.

*Pseudanthiashangapiko* sp. nov.	Holotype CAS 247252	Paratype USNM 443821	Paratype CAS 247254
Standard length (mm)	45.2	28.1	33.2
Head length	30.8	33.3	31.4
Greatest body depth	27.6	26.3	29.1
Body width	14.2	10.8	14.8
Snout length	7.3	8.9	9.2
Bony interorbital width	7.5	9.1	8.7
Orbit diameter	7.8	10.9	9.4
Upper jaw length	12.9	15.7	14.8
Maxilla width	3.9	4.8	5.6
Caudal peduncle length	11.7	10.0	10.1
Caudal peduncle depth	12.7	12.8	14.2
Predorsal length	29.8	31.1	35.1
Preanal length	55.6	65.1	64.8
Prepelvic length	33.8	34.7	30.8
Dorsal fin base length	57.9	55.7	61.6
First dorsal spine	4.9	5.0	4.3
Longest dorsal spine (number)	13.4 (4)	16.4 (3)	15.2 (4)
First segmented dorsal ray	12.7	16.7	14.6
Longest segmented dorsal ray (number)	15.6 (4)	18 (4)	15.7 (4)
Anal fin base length	21.6	18.0	19.2
First anal spine	6.2	4.9	4.8
Second anal spine	13.4	13.6	12.1
Third anal spine	12.6	11.6	9.9
First segmented anal ray	13.6	13.8	14.0
Longest segmented anal ray (number)	20.4 (4)	15.5 (4)	15.3 (4)
Caudal fin length	32.4	25.0	26.4
Pectoral fin length	26.3	29.7	28.1
Pelvic spine length	18.3	19.7	17.1
Pelvic fin length	31.1	26.8	23.7

Mitochondrial cytochrome c oxidase subunit I (COI) DNA was sequenced and analyzed for the new species. DNA extraction and PCR amplification of the COI were performed following protocols described by Arango et al. (2019), using BOLFishF1/BOLFishR1 primers. Alignments of DNA sequences were done using a standard Geneious global alignment with free end gaps and 65% similarity in the program Geneious Prime 2020.0.3 (Kearse et al. 2012). Because taxon sampling for *Pseudanthias* in public databases and our lab is not sufficient for a detailed phylogenetic analysis, and we only sequenced one mitochondrial DNA marker, we did not attempt a phylogenetic reconstruction. For our comparisons, genetic distances are uncorrected. COI sequences were compared to sequences of all other available Anthiadinae in GenBank, which include 29 species of *Pseudanthias* as well as representatives of the following genera: *Anthias*, *Baldwinella*, *Choranthias*, *Hemanthias*, *Luzonichthys*, *Meganthias*, *Nemanthias*, *Odontanthias*, *Plectranthias*, *Sacura*, *Serranocirrhitus*, and *Tosanoides*. The GenBank accession numbers for *Pseudanthiashangapiko* sp. nov. are MZ087699, MZ087670, and MZ087671. The holotype is deposited in the California Academy of Sciences ichthyological collection (CAS 247252) and the paratypes are deposited at the California Academy of Sciences ichthyological collection (CAS 247254) and the Smithsonian National Museum of Natural History (USNM 443821).

## Taxonomy

### Pseudanthias hangapiko sp. nov.

Taxon classificationAnimaliaPerciformesSerranidae

622C913C-E9EE-5C74-AB52-AB1EFA0193AC

http://zoobank.org/4F39F82B-8465-4694-9A4E-A674D800C33E

**Figure 1.**
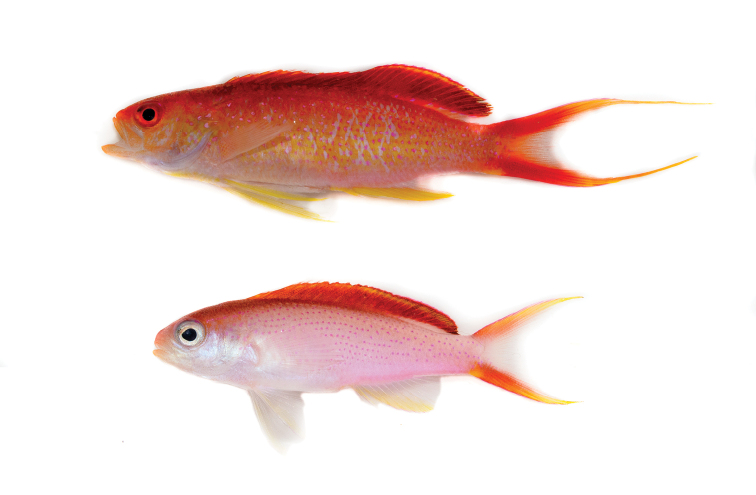
Holotype CAS 247252, male specimen (upper), 45.2 mm SL. Paratype CAS 247254, female specimen (lower), 33.2 mm SL. Photos by LA Rocha.

**Figure 2.**
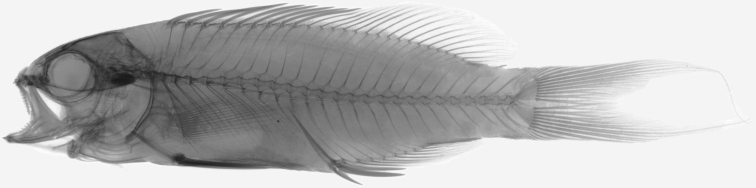
Radiograph of holotype, CAS 247252. Photo by J Fong.

**Figure 3.**
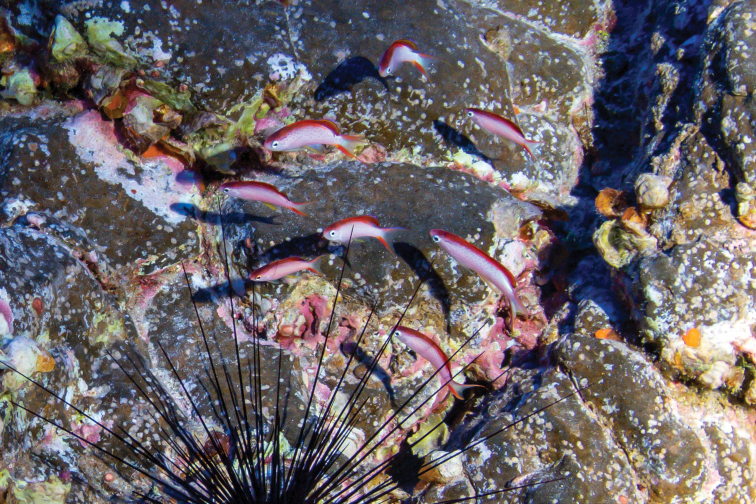
An aggregation of *Pseudanthiashangapiko* sp. nov. on a rocky mesophotic coral ecosystem at Rapa Nui (Easter Island) at 80 m depth.

Figs 1 , 2 , 3 , Table 1

#### Type locality.

Hanga Piko, Rapa Nui (Easter Island), Chile ***Holotype*.** CAS 247252 (Field number LAR2642). Male, 45.2 mm SL, GenBank accession number MZ087699. Location: Hanga Piko, Rapa Nui, Chile (27°9'12"S, 109°26'52"W). Collected by B. Shepherd, L.A. Rocha, T.A.Y. Phelps, and M.V. Bell using a hand-net at 83 m depth, 7 March 2017 (Figs 1, 2). ***Paratypes*.** USNM 443821 (Field number LAR2643). Male, 28.1 mm SL, GenBank accession number MZ087700. CAS 247254 (Field number LAR2645). Female, 33.2 mm SL, GenBank accession number MZ087701. Both from the same location as the holotype. Collected by B. Shepherd, L.A. Rocha, T.A.Y. Phelps, and M.V. Bell using a hand-net, 7 March 2017 (Fig. 1).

#### Diagnosis.

The following combination of characters distinguishes *Pseudanthiashangapiko* sp. nov. from congeners: dorsal rays X, 17; anal rays III, 8; pectoral rays 16 (left side of one specimen 17); vertebrae 10+16; scales relatively large, two scales between lateral line and base of fifth dorsal spine, and 16 (17) circumpeduncular scales; gill rakers 11+22–23; body very slender and compressed, the greatest body depth 3.4–3.8 in SL; caudal peduncle short, its length 2.6–3.3 in HL; sexually dichromatic, with male coloration red dorsally, yellow laterally, silvery-pink on throat and belly; females pink, silvery-pink on operculum, throat and belly; both sexes dark red on top of head, along anterior two-thirds of dorsal fin base; both sexes with rows of irregularly-spaced metallic magenta spots laterally, and red dorsal and caudal fins with yellow highlights.

#### Description.

Dorsal fin X, 17; anal fin III, 8; pectoral rays 16 (one paratype with 17 rays on left pectoral fin), upper two and lowermost unbranched; pelvic fin I, 5; principal caudal-fin rays 9 + 8 (7 + 6 branched); upper procurrent caudal-fin rays 9 (10); lower procurrent caudal rays 9 (9, 10?); tubed lateral-line scales 40 | 41 (40–43); scales above lateral line to origin of dorsal fin 5 (4); scales above lateral line to base of fifth dorsal spine 2; scales below lateral line to origin of anal fin 11 (12); circumpeduncular scales 16 (17); gill rakers 11+23 (11+22–23); pseudobranchial filaments 10 (9); branchiostegal rays 7; vertebrae 10+16; supraneurals 2; predorsal formula 0/0/2/1+1; main shaft (proximal component) of first dorsal pterygiophore inclined slightly backwards; dorsal pterygiophores in interneural spaces 9–13 1/1/1+1/1+1/1; terminal dorsal pterygiophore in interneural space 19 (18); no trisegmental pterygiophores associated with dorsal fin; proximal tip of first anal-fin pterygiophore near distal tip of haemal spine on first caudal vertebra; terminal anal pterygiophore in interhaemal space 6; no trisegmental pterygiophores associated with anal fin; ribs present on vertebrae 3 through 10; epineurals present on vertebrae 1 through 13 (12?); no paired parapophyses on first caudal vertebra; parhypural and hypurals autogenous; well-developed hypurapophysis on parhypural; epurals 3; single uroneural (posterior uroneural absent); ventral tip of cleithrum with well-developed posteroventral process.

Body very slender, compressed, its depth 3.6 (3.4–3.8) in SL, the width 2.0 (2.0–2.4) in depth; head length 3.2 (3.0–3.2) in SL; snout length 4.2 (3.4–3.7) in HL; snout and front of upper lip rounded, lacking fleshy anterior extension; diameter of orbit 3.9 (3.1–3.3) in head; posterior edge of orbit with 13 (12) fleshy papillae; interorbital space smooth, the bony width 4.1 (3.6–3.7) in HL; least depth of caudal peduncle 2.4 (2.2–2.6) in HL; caudal peduncle length 2.6 (3.1–3.3) in HL.

Mouth moderately large, slightly oblique, the posterior margin of the maxilla reaching a vertical through the center of the pupil; lower jaw does not protrude when mouth is closed; maxilla width 2.0 (1.7–2.3) in orbit diameter. Upper jaw with two pairs of slightly enlarged canines directed ventrally; a band of small conical teeth, three rows wide at symphysis, reducing to two rows on sides of jaw, with the outer row teeth much larger and slightly curved dorsally, and the inner pair of teeth anteriorly nearest symphysis enlarged and caniniform; dentary with two rows of small conical teeth narrowing to one row and becoming larger posteriorly; lower jaw with one to two enlarged, curved, forward-projecting canine teeth on either side of symphysis; vomer with triangular patch of small conical teeth; palatine with a narrow band of small conical teeth, five rows wide, decreasing to one row posteriorly; tongue small, triangular, pointed, and edentate.

Anterior nostril positioned at middle of snout, with a short fleshy flap on posterior margin; posterior nostril at mid-upper anterior border of orbit, covered by a thin, narrow membrane anteriorly. Opercle with three flat spines, all stout and acute; the middle opercle spine largest and level with center of eye; the upper smallest; ventral margin of preopercle smooth; vertical margin of preopercle with 14 acute spines (11), the largest almost the same size as the inferior opercle spine; posterior margin of subopercle with two strong spines; posterior corner of interopercle with one strong, acute spine.

Scales ctenoid, relatively large, without basal cteni; head and preopercle scaled; distal portion of maxilla covered with scales, head fully scaled except for lips and areas in front of and immediately below nostrils; dorsal fin and anal fin without scales; proximal one-third of pelvic fin scaled; caudal fin with scales extending approximately three quarters distance to posterior margin; scales cover the central portion of the proximal one-fifth of the pectoral fin. Lateral line complete, smoothly curved, mostly follows dorsal contour of body reaching its highest point below the fifth dorsal spine.

Origin of dorsal fin at vertical through base of pectoral fin, the predorsal length 3.4 (2.8–3.2) in SL; first dorsal spine 6.3 (6.6–7.2) in HL; fourth dorsal spine longest (third in smaller paratype), 2.3 (2.0–2.1) in HL; first dorsal ray 2.4 (2.0–2.2) in HL, longest dorsal ray the fourth, 2.0 (1.8) in HL; origin of anal fin below base of third dorsal soft ray, the preanal length 1.8 (1.5) in SL; first anal spine 5.0 (6.6–6.8) in HL; second anal spine the longest, nearly three times the length of the first, 2.3 (2.5–2.6) in HL; third anal spine 2.4 (2.9–3.2) in HL; posterior margin of anal fin rounded, the first segmented ray 1.4 (2.4–3.2) in HL, the longest segmented ray the fourth, 1.5 (2.0–2.1) in HL. Caudal fin lunate with trailing filaments, longer in males, the caudal concavity 3.1 (3.8–4.0) in SL. Pectoral fins 3.8 (3.4–3.6) in SL, extending to a vertical below base of first dorsal soft ray. Pelvic fins moderately long, 5.4 (5.1–5.9) in SL reaching second anal spine.

***Color in life*:***Pseudanthiashangapiko* sp. nov. is sexually dichromatic. **Males** (Fig. 1): body pink, with yellow and dark red obscuring most of the ground color, except on belly and throat. Rows of metallic magenta spots, about one per scale, cover body, creating an irregularly spotted pattern starting from behind orbit and extending to base of caudal fin; upper third of body dark red, sides yellow. Dorsal fin dark red, with thin yellow stripe following upper margin; posterior half of soft dorsal-fin base with region of less-pronounced color, extending approximately one quarter of the height of dorsal fin; dark gray region on upper posterior margin of dorsal fin, spanning last five to six fin rays; pectoral fins hyaline; pelvic and anal fins yellow on anterior half, hyaline posteriorly; caudal fin red with yellow-orange filaments; yellow patch at ventral origin of caudal fin. Head pale orange, red along snout and between eyes, operculum yellow. Eye red, darker along outer edge. **Females** (Figs 1, 3): body predominantly pink with less-pronounced red and yellow markings, silvery-pink on operculum, throat and belly; spotting pattern of metallic magenta scales more widely-spaced than in males; anterior dorsal third of head dark red, pale silvery-pink below; faint orange on tip of snout and lower jaw. Dorsal fin red, with thin yellow stripe following upper margin, region of lighter color on posterior half of soft dorsal-fin base more pronounced in females than in males, same pink as body ground color; dark gray region on upper margin of dorsal fin more pronounced than in males, spanning posteriormost ten to twelve fin rays; pectoral fins hyaline; pelvic and anal fins hyaline with faint yellow markings anteriorly, thin magenta line on distal edge of anal fin; outermost caudal-fin rays yellow with red, especially on upper and lower margins and near base of caudal fin, centermost caudal-fin rays hyaline distally, thin dark gray lines on distal edges of upper and lower caudal fin filaments. Eye silver, darker along outer edge.

***Color in alcohol*:** All specimens straw-colored, except dorsally, where all specimens are darkly pigmented above lateral line where red in life; dorsal fin translucent with dark pigment, all other fins translucent.

#### Etymology.

The species is named for the location where it was collected, Hanga Piko, meaning “hidden bay” in the Rapa Nui language. To be treated as a noun in apposition.

#### Common name.

Rapa Nui Fairy Basslet.

#### Distribution and habitat.

The new species is currently known only from Rapa Nui. The holotype and paratypes were collected at a depth of 83 m at a small, rocky patch reef surrounded by a large sandy area (Fig. 3). Other species collected at this location and recently described by our team include *Plectranthiasahiahiata*, *Luzonichthyskiomeamea*, and *Chromismamatapara* (Shepherd et al. 2018, 2019, 2020). Due to geographical isolation and the high degree of endemism (21.7%) among the shore fishes of Rapa Nui (Delrieu-Trottin et al. 2019), it is likely that *Pseudanthiashangapiko* sp. nov. is endemic to the island.

#### Remarks.

Coloration is important for the identification of *Pseudanthias* (Randall and Pyle 2001). *Pseudanthiashangapiko* sp. nov. is distinguished from all congeners in coloration of adult individuals: males with red and yellow mostly obscuring a pink ground color; females mostly pink; both sexes silvery-pink on throat and belly, with rows of irregular metallic magenta spots, a dark red region along the anterior-dorsal portion of the body, and predominantly red and yellow dorsal and caudal fins. In morphology, *Pseudanthiashangapiko* sp. nov. most resembles *Pseudanthiasconnelli* Heemstra & Randall, 1986, *Pseudanthiasrandalli* Lubbock & Allen, 1978, and *P.squamipinnis*, sharing overlapping counts in the number of spines and rays on the dorsal and pectoral fins, the number of lateral line scales, and the number of gill rakers. However, it can be differentiated from all of these species by coloration and morphology, especially in the number of segmented rays on the anal fin (8, vs. 7 in *P.connelli*, *P.randalli*, and *P.squamipinnis*), by having a very slender body (greatest body depth 3.4–3.7 in SL), and in the small number of circumpeduncular scales (16–17, vs. 22–25 in *P.connelli*, *P.randalli*, and *P.squamipinnis*). *Pseudanthiashangapiko* sp. nov. lacks the scaly dorsal and anal fins and auxiliary scales present in *P.squamipinnis*, and has only two supraneurals, not three as in *P.squamipinnis*. Male *Pseudanthiashangapiko* sp. nov. lack the pennant-like extension on the third dorsal spine occurring in *P.squamipinnis*, and male *Pseudanthiashangapiko* sp. nov. do not possess a fleshy, protruding upper lip, present in the subgenus Mirolabrichthys, but rather have a rounded snout. It should be noted that the diagnostic validity of this latter character is questionable, as some species exhibit various degrees of hypertrophy and the character may have risen independently multiple times within the anthiadine fishes (Gill et al. 2017).

The most similar DNA barcodes (mitochondrial COI gene) are from *Pseudanthiasventralis* and *P.hawaiiensis*, with 16.8% and 17.0% uncorrected divergence, respectively. These distances are much higher than average divergences between sister species (Rocha 2004), and even between genera, so it is not surprising that *P.hangapiko* can be differentiated from these two species by several characters, including the number of anal-fin rays (III, 8 vs. III, 9–10 in *P.ventralis* and *P.hawaiiensis*), body depth (3.4–3.8 vs 2.3–3.0), and also by body coloration.

## Discussion

The presence of *Pseudanthiashangapiko* sp. nov. in Rapa Nui extends the known geographic range of the genus *Pseudanthias* by nearly 2,000 km eastward in the South Pacific. *Pseudanthiasventralis*, the species with the smallest uncorrected genetic distance at the mtDNA COI gene, is also the one occurring nearest, with the south-easternmost edge of its range occurring at Pitcairn Island (Randall 1979). Although the type specimens for *P.ventralis* are from Pitcairn, it likely comprises a species flock with at least three distinct groupings based on coloration and geographical range (Kuiter 2004), and many phylogeographic studies have found evidence of strong biogeographic partitions in the Pacific resulting in genetically distinct conspecifics (Bowen et al. 2016). A closely related species, *P.hawaiiensis*, marks the north-easternmost edge of the genus’ distribution in Hawai’i (Randall 1979). Not surprisingly, diversity within the genus *Pseudanthias* decreases moving eastward out of the coral triangle: there are at least 30 species of *Pseudanthias* in the Coral Triangle (Allen and Erdmann 2012), however, aside from *P.ventralis*, only an additional nine species of *Pseudanthias* occur in French Polynesia (Bacchet et al. 2017). Only three species, all presumed to be endemic, occur in the Marquesas (Williams et al. 2013; Delrieu-Trottin et al. 2015). There are no *Pseudanthias* known from the Eastern Pacific, which is relatively well-explored even at depth (Cortés 2019). As such, *Pseudanthiashangapiko* sp. nov. likely marks the easternmost distribution for the genus.

Taxonomy within the anthiadine fishes is problematic. Several genera, including *Pseudanthias*, may be well-documented and easily recognized groups of reef fishes, but they are poorly diagnosed, likely polyphyletic, and greatly in need of revision (Gill et al. 2017). With more complete sampling of extant taxa using multiple molecular markers, several members of the genus *Pseudanthias* will likely be assigned to other genera (Gill et al. 2017). Additionally, the high genetic distance between *P.hangapiko* and its closest sequenced relatives (>16%) indicates that the placement of this new species within the genus *Pseudanthias* is uncertain. Nevertheless, creating new genera without a complete understanding of the phylogenetic and morphological variation within *Pseudanthias* today would likely only generate more taxonomic instability. Thus, the generic placement of *P.hangapiko* should be considered provisional.

Understanding remote island ecosystems is critical to advancing scientific knowledge of speciation and ecology (Pinheiro et al. 2017; Quimbayo et al. 2019). However, the challenging logistics of conducting mesophotic exploration in geographically isolated locations such as Rapa Nui has undoubtedly limited knowledge of the deep reef biodiversity occurring in such places. With recent advances in technology, such as remote operated vehicles (ROV), consumer-grade closed-circuit rebreathers (CCR), baited remote underwater video (BRUV) employing inexpensive, low-light digital cameras, and autonomous underwater vehicles (AUV), scientists now have multiple tools to use for exploring deeper habitats that were not available to us even a decade or two ago (Pimentel et al. 2020). These studies remain expensive and administratively complex, requiring both significant funding and effective collaboration in order to be successful. Our 2017 expedition to Rapa Nui resulted in descriptions of four new species, all of which were collected on a pair of dives at a single location near the island. This remarkable discovery rate demonstrates the magnitude of the biodiversity waiting to be unveiled in Rapa Nui and elsewhere through deep reef exploration and purposeful collaboration.

## Supplementary Material

XML Treatment for Pseudanthias hangapiko
